# A short artificial antimicrobial peptide shows potential to prevent or treat bone infections

**DOI:** 10.1038/s41598-017-01698-0

**Published:** 2017-05-04

**Authors:** N. Bormann, A. Koliszak, S. Kasper, L. Schoen, K. Hilpert, R. Volkmer, J. Kikhney, B. Wildemann

**Affiliations:** 1 Julius Wolff Institute, Charité - Universitätsmedizin Berlin, Berlin, Germany; 2 Berlin-Brandenburg Center for Regenerative Therapies, Charité-Universitätsmedizin Berlin, Berlin, Germany; 3 Centre for Musculoskeletal Surgery, Charité - Universitätsmedizin Berlin, Berlin, Germany; 4 Institute of Infection and Immunity, St George’s University of London, London, United Kingdom; 5 Institute for Medical Immunology, Charité - Universitätsmedizin Berlin, Berlin, Germany; 6 Institute for Microbiology and Hygiene, Charité – Universitätsmedizin Berlin, Berlin, Germany; 7 Biofilmcenter, German Heart Institute Berlin, Berlin, Germany; 8 Leibniz-Institut für Molekulare Pharmakologie, Berlin, Germany

## Abstract

Infection of bone is a severe complication due to the variety of bacteria causing it, their resistance against classical antibiotics, the formation of a biofilm and the difficulty to eradicate it. Antimicrobial peptides (AMPs) are naturally occurring peptides and promising candidates for treatment of joint infections. This study aimed to analyze the effect of short artificial peptides derived from an optimized library regarding (1) antimicrobial effect on different bacterial species, (2) efficacy on biofilms, and (3) effect on osteoblast‑like cells. Culturing the AMP-modifications with *Escherichia coli, Enterococcus faecalis*, *Pseudomonas aeruginosa, Staphylococcus aureus* (including clinical isolates of *MRSA* and *MSSA*) and *Staphylococcus epidermidis* identified one candidate that was most effective against all bacteria. This AMP was also able to reduce biofilm as demonstrated by FISH and microcalorimetry. Osteoblast viability and differentiation were not negatively affected by the AMP. A cation concentration comparable to that physiologically occurring in blood had almost no negative effect on AMP activity and even with 10% serum bacterial growth was inhibited. Bacteria internalized into osteoblasts were reduced by the AMP. Taken together the results demonstrate a high antimicrobial activity of the AMP even against bacteria incorporated in a biofilm or internalized into cells without harming human osteoblasts.

## Introduction

Infections of bone are a severe complication and can result in a long-term treatment. Infected/contaminated material (tissue and implant) must be removed and the infection cleared. *Staphylococcus aureus* is the predominant bacteria identified in fracture-fixation device associated infections (30%) followed by coagulase-negative staphylococci (22%), but also Gram-negative bacteria, enterococci, streptococci and other species may be isolated^1^. The interaction of *S*. *aureus* with bone or especially osteoblasts is well investigated^2^. For example, *S*. *aureus* expresses several molecules (collagen adhesin, bone sialoprotein binding protein, MHC II analog protein) that support the binding with proteins of the extracellular matrix of bone, but also induces the secretion of inflammatory factors and chemokines, and can induce apoptosis. A mechanism that allows *S*. *aureus* to persist protected from the immune system and most antibiotics is the internalization into host cells, especially osteoblast^3^. Within the last decades the number of resistant bacteria increased, resulting in more challenges for the treatment. Facing the impact of resistant bacteria on the human health and the health care systems, antimicrobial resistance (AMR) is identified as one of the major health problems^4^ resulting in the endorsement of a global action plan by the World Health Organization to tackle antimicrobial resistance. Besides the need to increase the awareness to this problem and the improvement of the antibiotic use, new antimicrobial active drugs must be developed. An interesting group of antimicrobial active drugs are antimicrobial peptides (AMP). AMPs are widely found in nature and more than 2700 different AMPs are described so far [http://aps.unmc.edu/AP/main.php]^5^. As part of the innate immune system, the main task of AMPs is to build up the first defense barrier against entering pathogens. They are approximately between 10–50 amino acids in length and mostly cationic amphipathic peptides, which can interact with the bacterial cell membrane. In recent years it became clear that they have different modes of actions, besides the “classical” one to cause cell lysis of bacteria^6^. AMPs act against Gram-positive and -negative bacteria, viruses and fungi, they do not easily induce resistance and can successfully attack bacteria within a biofilm^7–11^. Due to their interaction with the cell membrane, which is beneficial for their antimicrobial activity, they might also interfere with eukaryotic cell membranes resulting in unwanted side effects in therapeutic application. It was previously reported that the activity of AMPs is reduced due to albumin present in human serum^12, 13^ and divalent cations such as Ca²^+^ and Mg²^+ ^^12, 14^. It is therefore recommended to use a cation-reduced medium for microbiological test^15^. However, this will not represent the physiological condition in the human body. In this study, the properties of five synthetic peptides were investigated. The used peptides were created by peptide array technology as previously described^16–18^. The effect of five 9mer peptides regarding the killing of various bacteria was investigated and the most effective peptide was used for further studies. With this peptide the effect on bacteria in a biofilm, the possible cytotoxic effect on human osteoblast-like cells, and the killing of bacteria within osteoblast-like cells was analyzed.

## Results

### Antimicrobial activity

**Figure 1:**
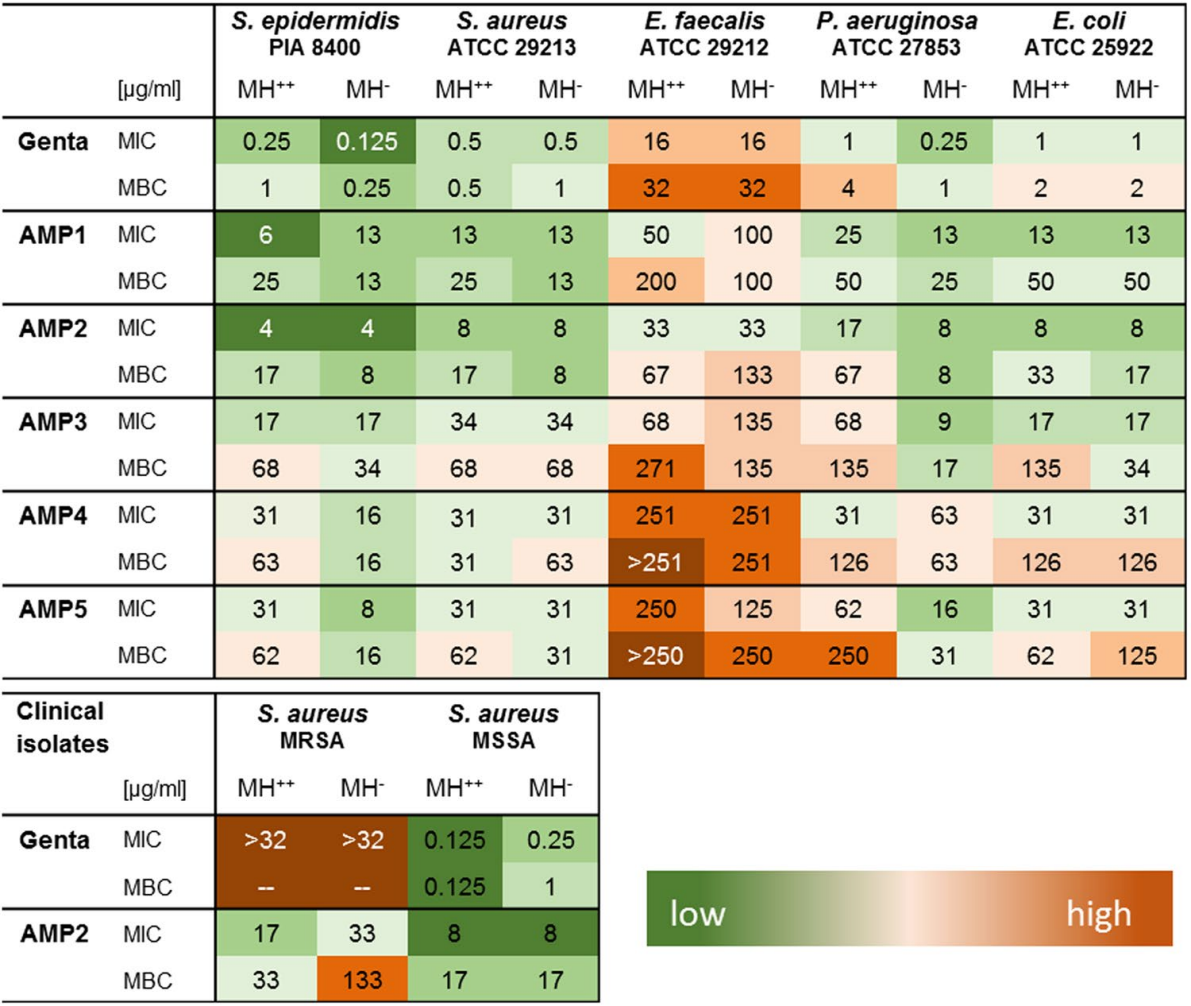
Minimal inhibitory concentration (MIC) and minimal bactericidal concentration (MBC) in µg/ml for gentamicin (Genta) and for five different AMPs. MH^++^: Mueller-Hinton bouillon with cations (n = 4–8), MH^−^: Mueller-Hinton bouillon without cations (n = 4). Color code: green low concentration, dark orange high concentration needed for antimicrobial effect.

The antimicrobial activity of five different AMPs was tested and gentamicin served as control. The inhibitory and bactericidal concentration was tested on seven Gram-positive or -negative bacterial strains (Fig. 1), two of them were clinical isolates. All tested AMPs inhibited bacterial growth at a concentration ≤251 µg/ml. Except for *E*. *faecalis*, all bacteria were killed with the tested concentrations. Testing the influence of cations on the activity of the peptides revealed no major differences between the tested media. In some cases, e.g. *P*. *aeruginosa*, the MIC of AMPs was 3–8 concentrations higher for the medium with cations (MH^++^) compared to the medium without cations (MH^−^). Using clinical isolates of *MRSA* and *MSSA*, the AMP2 inhibited growth and was bactericidal, whereas gentamicin showed no growth inhibition of the *MRSA* as expected.

In a further experiment the possible effect of serum on AMP activity was investigated. AMP2 (67 µg/ml) was preincubated for 2 hours at 37 °C with different concentrations of FCS and then added to *S*. *aureus*. A dosage dependent inhibition of the AMP activity was detected (R^2^ = 0.863 with p = 0.000012), but even with 10% FCS the bacterial growth was less than 2% compared to the group without AMP (Table 1).

**Table 1:** Effect of FCS on AMP2 (67 µg/ml) activity.

	w/o AMP (0% FCS)	0% FCS	0.1% FCS	1% FCS	10% FCS
cfu/ml ± StD	141,111 ± 86,111	7 ± 9	37 ± 13	553 ± 255	2,078 ± 540

Shown are the results of bacterial growth in cfu/ml ± standard deviation (n = 3). Data are shown as means with standard deviation. Regression analysis: R^2^ = 0.862 with p = 0.000012.

### Effect on osteoblast like cells

**Figure 2:**
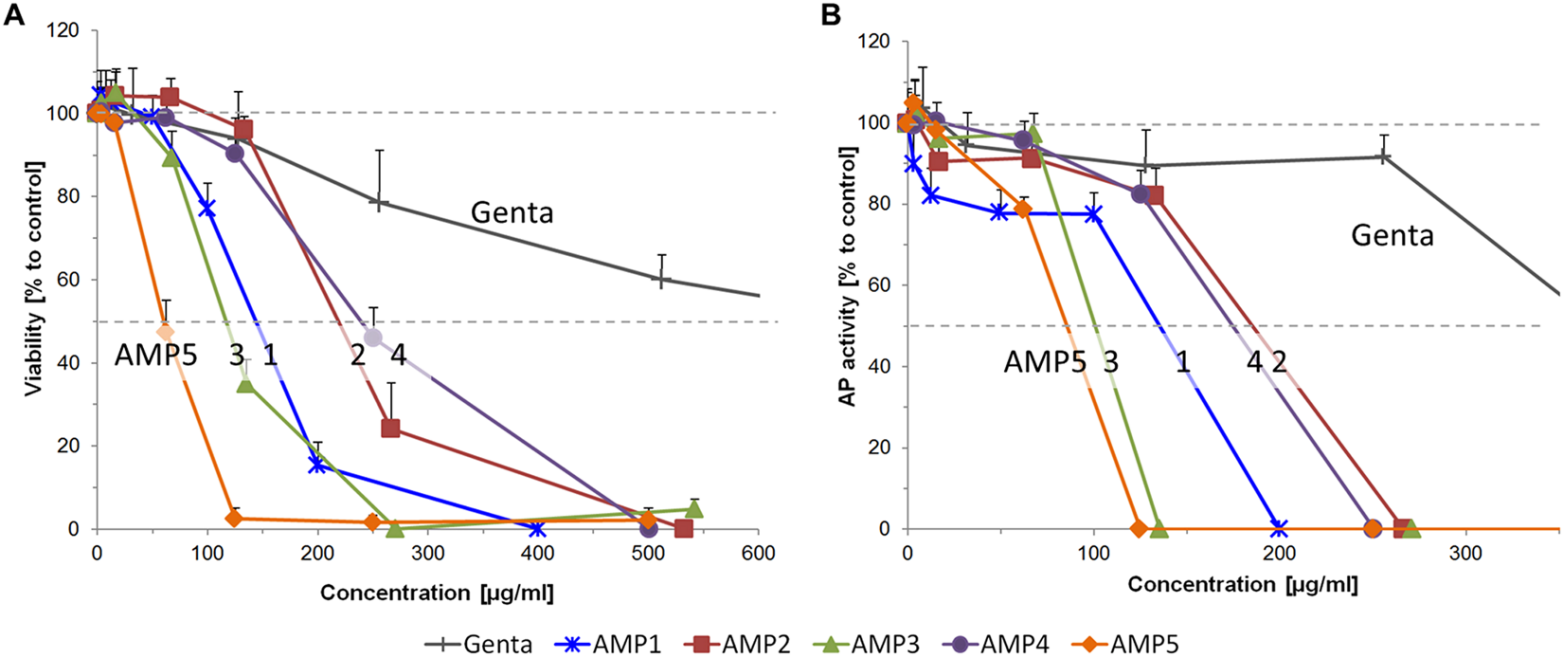
Human primary osteoblast-like cells were cultured with different concentrations of AMP and gentamicin for three days. Viability **(A)** and alkaline phosphatase (AP) activity **(B)** were determined. Values for AP were normalized to 10^4^ cells and compared to control (untreated cells, 100%). Results are means of n = 6, cells from two donors, triplicates.). Data are shown as means with standard deviation. Kruskal-Wallis, Mann-Whitney, Bonferroni-Holm post-hoc test. Significant inhibition compared to the control group, viability: AMP1 ≥ 100 µg/ml, AMP2 ≥ 267 µg/ml, AMP3 ≥ 68 µg/ml, AMP4 ≥ 251 µg/ml, AMP5 ≥ 63 µg/ml; Alkaline phosphatase activity: AMP1 all concentrations, AMP2–4 ≥ 125 µg/ml, AMP5 ≥ 63 µg/ml.

All five AMPs were investigated regarding their possible cytotoxic effect on primary human osteoblast-like cells. As initial outcome parameter, cell viability was determined after incubation with different concentrations of the AMPs or gentamicin. The lower concentrations (AMP1 < 100 µg/ml, AMP2 < 267 µg/ml, AMP3 < 68 µg/ml, AMP4 < 251 µg/ml, AMP5 < 63 µg/ml) of the AMPs had no negative effect on cell viability, whereas the higher concentrations reduced cell viability significantly (p < 0.005) (Fig. 2A).

Alkaline phosphatase (AP) activity was determined after three days. Again, the low concentration of the AMP had no negative effect (except of AMP1, which reduced significantly AP activity at all concentrations), while with increasing AMP or gentamicin concentrations, AP activity was decreased. For AMP2-4, only the last three concentrations (125–541 µg/ml) reduced significantly the AP activity (p < 0.005) (Fig. 2B).

Because AMP2 was antimicrobial active at concentrations that had no negative effect on the viability and AP- activity of the osteoblast-like cells, this peptide was used for further studies.

**Figure 3:**
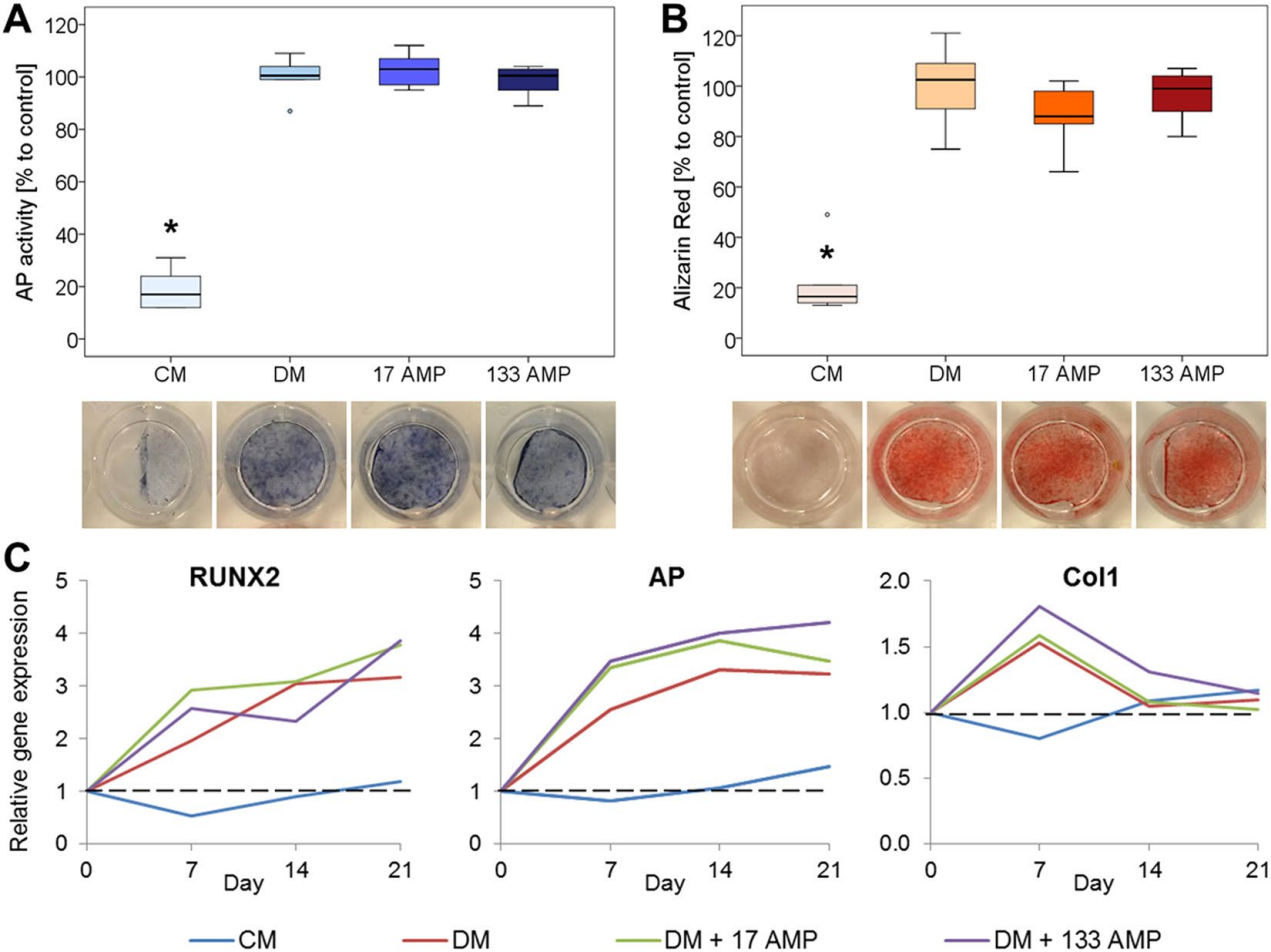
The differentiation experiment of the osteoblast-like cells was done with cultivation medium (CM), differentiation medium (DM) and DM with low (17 µg/ml) and high (133 µg/ml) AMP2. After 21 days alkaline phosphatase (AP) activity assay and staining **(A)** and alizarin red staining and quantification **(B)** were done (n = 6, cells from two donors, triplicates). Results were normalized to 10^4^ cells and expressed in percentage to control (DM) which was set 100%. Data are shown as median with 25 and 75 percentiles. Kruskal-Wallis, Mann-Whitney, Bonferroni-Holm post-hoc test: *Significant difference compared to the DM group, p ≤ 0.05**. (C)** Gene expression at days 0, 7, 14 and 21. Results are calculated with the ΔΔCt method with efficiency correction and normalization to the control (day 0 of differentiation) and 18 S ribosomal RNA used as a reference gene (n = 2). Data are shown as means with standard deviation.

The differentiation of the osteoblast-like cells was not affected by the low and high AMP concentration (17 + 133 µg/ml). There was no difference in the alkaline phosphatase activity between the groups cultivated with differentiation medium (DM) with or without AMP (Fig 3A). Cells in the cultivation medium (CM) showed only 20% AP activity compared to the control (DM without AMP). The mineralization assessed with alizarin red resulted in a deep red staining for the three DM groups (with and without AMP) and a faint staining for the CM group, which could be also quantitatively measured (Fig. 3B).

In addition, no effect on the osteogenic differentiation was observed for AMP2, as assessed by the expression of the markers runt-related transcription factor 2 (RUNX2), alkaline phosphatase (AP) and collagen type I alpha 1 (Col1).The genes RUNX2 and AP were upregulated in all groups cultivated with DM with/without AMP but not for the CM group. Col1 showed an upregulation on day 7 for the three DM groups and decreased then again to the level of day 0. For the CM, no Col1 regulation could be observed (Fig. 3C).

**Figure 4:**
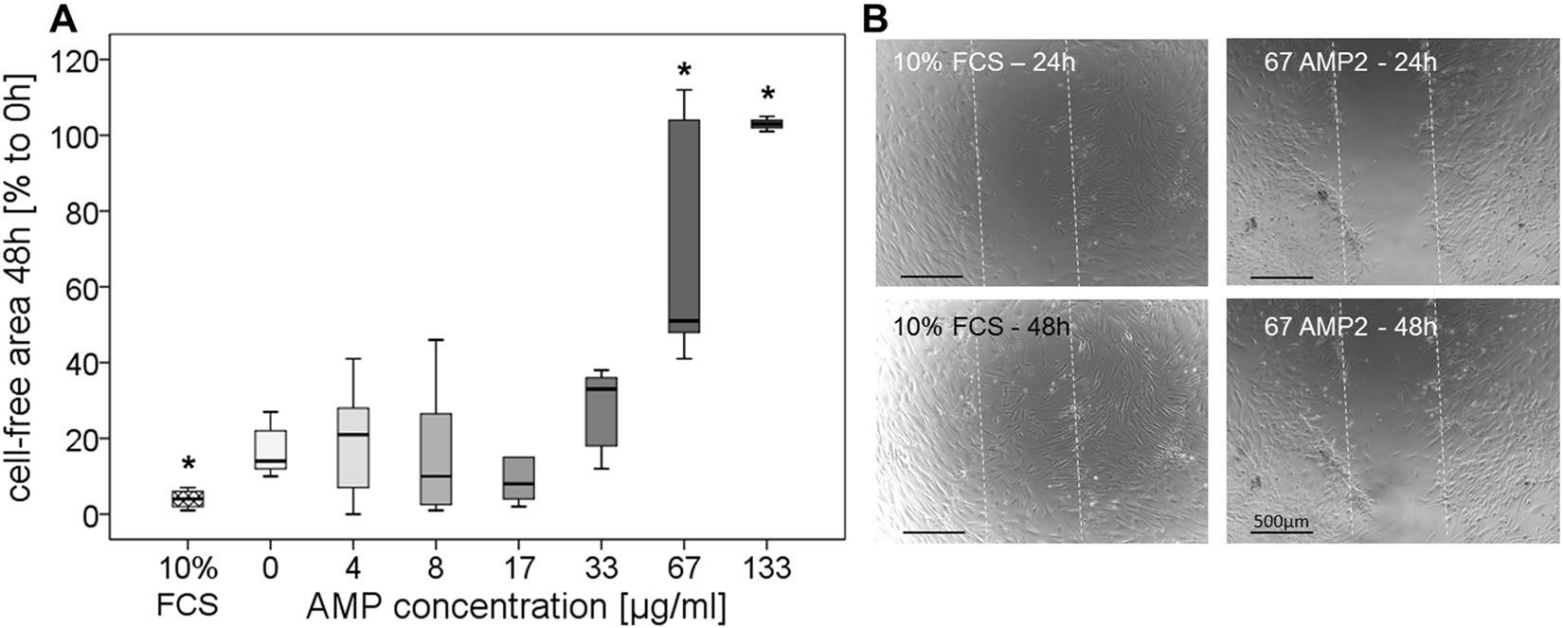
Scratch assay with human primary osteoblast-like cells and different AMP2 concentrations (0–133 µg/ml). Measurement of the gap was directly done after performing the scratch and after 24 and 48 hours incubation (n = 3–9, cells from one donor). (**A**) The cell-free area after 48 hours is shown in percentage of the gap compared to time point 0 hours ( = 100%). Positive control: CM with 10% FCS. Data are shown as median with 25 and 75 percentiles. Kruskal-Wallis, Mann-Whitney, Bonferroni-Holm post-hoc test: *Significant difference compared to group treated with 0 µg/ml AMP, p ≤ 0.05. (**B**) Exemplary pictures of positive control and 67 µg/ml AMP2 after 24 and 48 hours. The dotted lines mark the scratch. Scale bar 500 µm.

The possible effect of the AMP2 on migration was investigated in a scratch assay over 48 hours. After 24 hours, the positive control (Fig. 4B, 10% FCS – 24 h), the CM without and with 4–33 µg/ml AMP2 resulted in 50–66% free-area. AMP2 concentration of 67 µg/ml reduced the migration of cells (Fig. 4B, 67 AMP2 – 24 h) and with 133 µg/ml no migration was observed (data not shown). After 48 hours, a significant (p < 0.0001) closing of the gap could be seen for the positive control 10% FCS (Fig. 4B, 10% FCS – 48 h). AMP2 concentrations up to 33 µg/ml resulted in a cell-free area of 12–28%. With 67 µg/ml AMP2, the gap size was 71%, while it was still complete open using 133 µg/ml (Fig. 4A). With the high AMP2 concentration (67 and 133 µg/ml) cells started to change morphology, which might also be due to the low FCS content (0.1% FCS) (Fig. 4B, 67 µg/ml AMP2 – 48 h).

**Figure 5:**
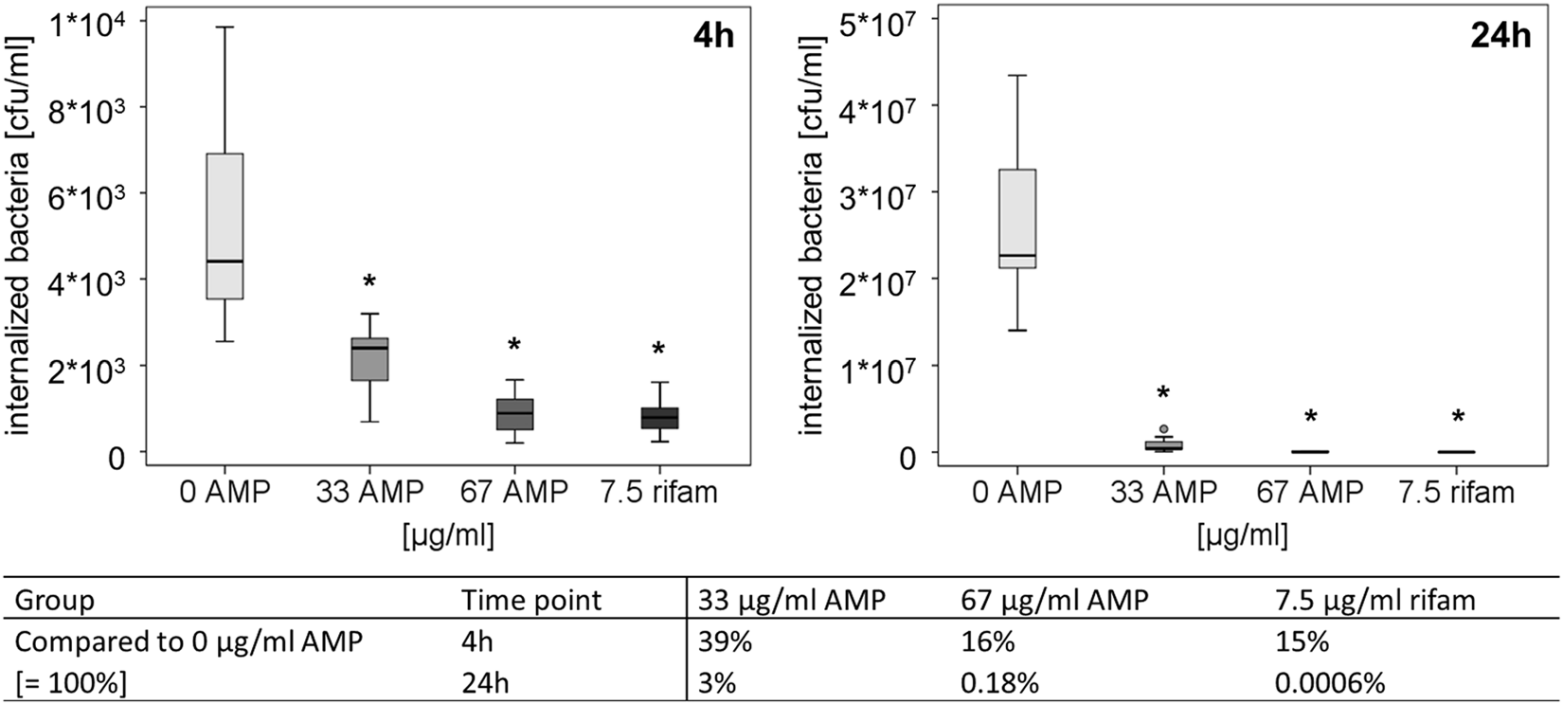
*S. aureus* internalized in human primary osteoblast-like cells were treated with 33 or 67 µg/ml AMP2 and 7.5 µg/ml rifampicin (rifam) for 4 hours and 24 hours (n = 7, cells from one donor). Data are shown as median with 25 and 75 percentiles. Kruskal-Wallis, Mann-Whitney, Bonferroni-Holm post-hoc test: *Significant compared to control group (0 µg/ml AMP), p ≤ 0.05.

The impact of AMP2 and rifampicin on internalized *S*. *aureus* was tested using human primary osteoblast-like cells. After 4 hours, no bacterial killing was seen in the control group (without AMP) and the bacteria continued to grow over 24 hours in the osteoblast-like cells. AMP2 (67 µg/ml) and rifampicin (7.5 µg/ml) decreased significantly the bacterial number after 4 hours and 24 hours. Cells treated with both AMP2 concentrations showed a bacterial load comparable to the starting point (0 hours). Compared to the untreated group, a strong inhibition of the bacteria was detectable (Fig. 5).

### Activity against biofilm

Two methods were used to investigate the effect of the AMP on biofilm. Using FISH, the biofilm was visualized and with microcalorimetry, the activity of the bacteria in the biofilm was quantified.

**Figure 6:**
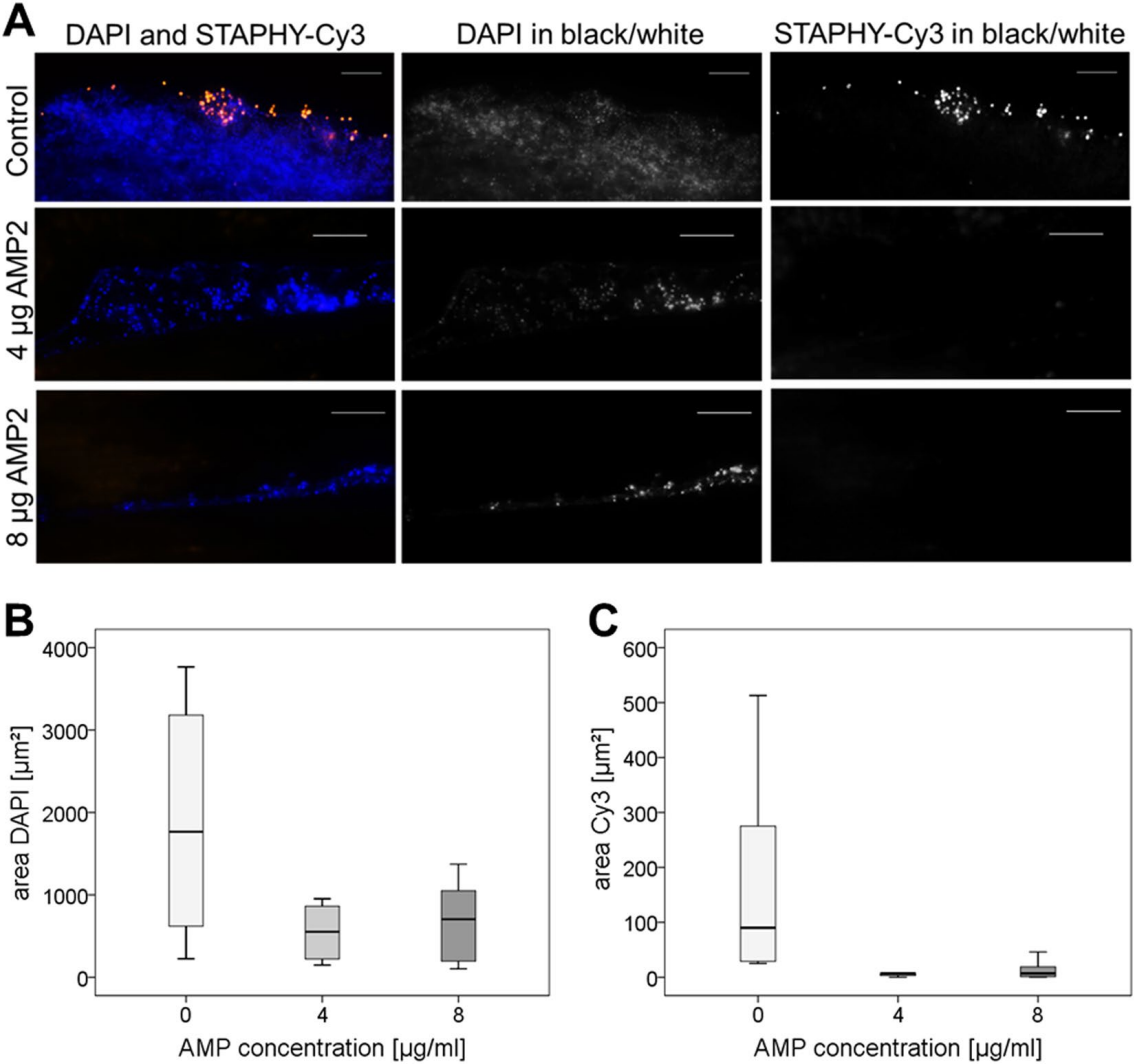
Fluorescence *in situ* hybridization (FISH) of *S. epidermidis* biofilms grown on polyurethane (PU) waver treated with 0, 4 or 8 µg/ml AMP2. (**A)** On the left hand, the panel shows the nucleic acid stain DAPI (blue) overlaid with the Cy3-labeled *Staphylococcus-*specific FISH-probe STAPHY (orange) for each group. The middle and right panels show either the separate DAPI or FISH Cy3 images in black and white of the respective colored image. AMP2 treatment led to a dramatic reduction of total biofilm area and fraction of FISH positive bacteria. Note also the altered fragmented appearance of the AMP treated biofilms. Scale bar = 10 µm. Pictures of 10 random areas per group were taken and results of digital images analysis are shown. **(B)** Total biofilm area (stained with DAPI). **(C)** Area of the FISH-positive bacteria (STAPHY-probe labelled with Cy3). n = 10 pictures per group. Data are shown as median with 25 and 75 percentiles.

Using FISH, *S*. *epidermidis* biofilms were microscopically evaluated over the whole lengths of the PU wavers (approximately 100 microscopic fields per waver) and showed upon AMP2 exposure an reduction in biofilm thickness and number of FISH-positive cells. In addition, the biofilm structure was altered (Fig. 6A). For a more objective assessment of the AMP2 effect the areas were quantified. The treatment with AMP2 resulted in an up to 76% reduction of the biofilm area (4 µg/ml) compared to the untreated group (Fig. 6B). The percentage FISH-positive (Cy3) area was reduced by 86% or 90% for the group treated with 4 or 8 µg/ml AMP2 (Fig. 6C).

In a further experiment the *S*. *aureus* growth was quantified over time by calorimetry. The MIC of AMP2 for planktonic *S*. *aureus* was 8 µg/ml for all three tested bouillons and 1 µg/ml for gentamicin (results were comparable to the broth microdilution method shown in Fig. 1).

**Figure 7:**
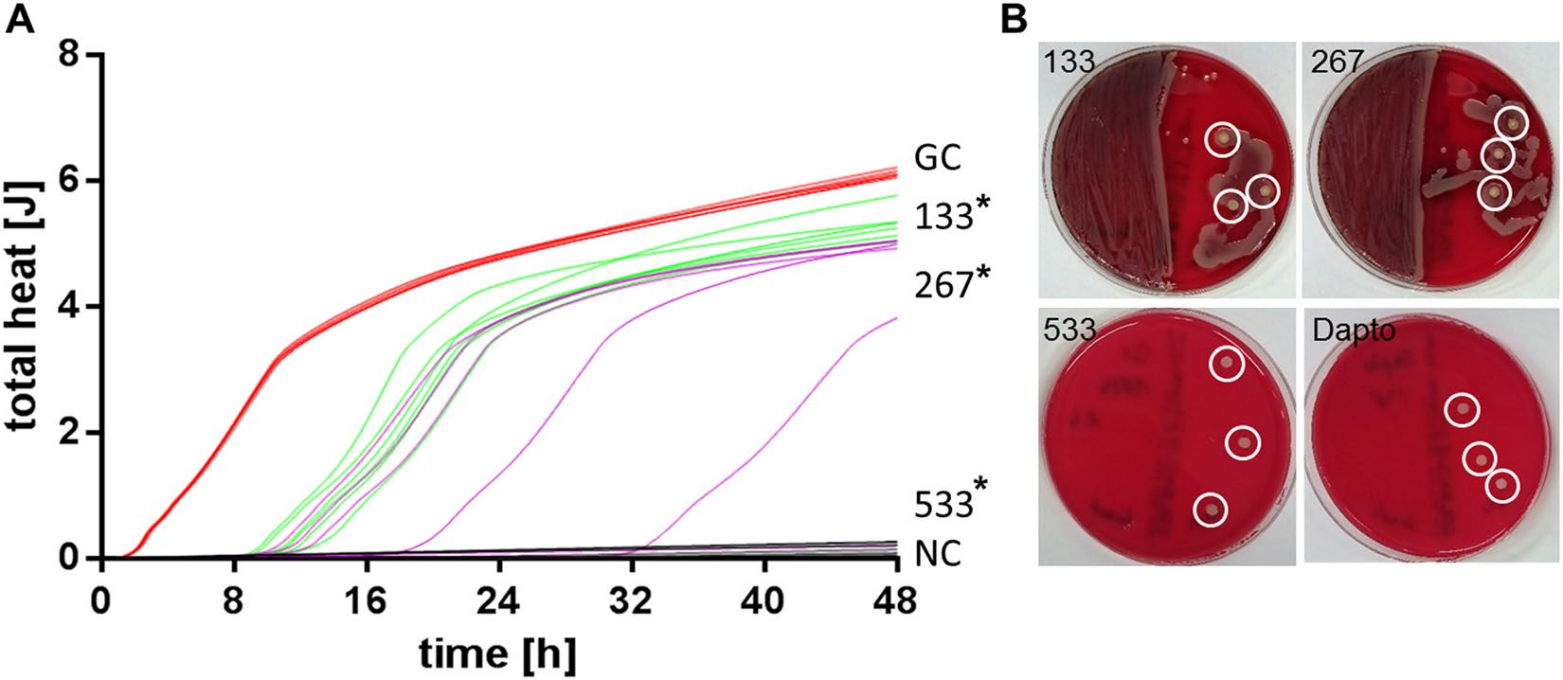
*S.aureus* biofilm was grown on glass beads and incubated with 133–533 µg/ml AMP2 and heat flow of the bacteria was measured over 48 hours. **(A)** Total heat over time. red: growth control (GC), gray: negative control (NC) - beads without bacteria, green: 133 µg/ml AMP2, purple: 267 µg/ml AMP2, black: 533 µg/ml µg/ml AMP2 (n = 6). Kruskal-Wallis, Mann-Whitney, Bonferroni-Holm post-hoc test: *Significant compared to growth control, p ≤ 0.05. **(B)** After calorimetry measurement, samples and glass beads were plated on one half of a CA plate, respectively. Shown are exemplarily plates with medium sample on the left and three glas beads (circles) of 133, 267, 533 µg/ml AMP2 and 128 µg/ml daptomycin on the right side.

To analyze the effect on the biofilm, AMP2 concentrations of 133–533 µg/ml were used and 128 µg/ml daptomycin served as control. Daptomycin completely inhibited the biofilm growth (data not shown), which could also be proven by plating the samples and glass beads on agar plates (Fig. 7B). After 48 hours, AMP2 concentration of 133 µg/ml significantly inhibited the biofilm by 13 ± 4% compared to the growth control. AMP2 concentration 267 µg/ml resulted in a 35 ± 29% inhibition and 533 µg/ml AMP2 in 97 ± 1%, which were both significant. The high standard deviation for the concentration 267 µg/ml is because one of the six samples completely killed the bacteria, whereas five of them only inhibited the biofilm by 18–38% (Fig. 7A).

The MBC_B_ was determined by plating samples and glass beads on the agar plates. All samples with 533 µg/ml AMP2 totally killed the *S*. *aureus* biofilm, as shown by no growth (Fig. 7B).

### Local drug delivery

**Figure 8:**
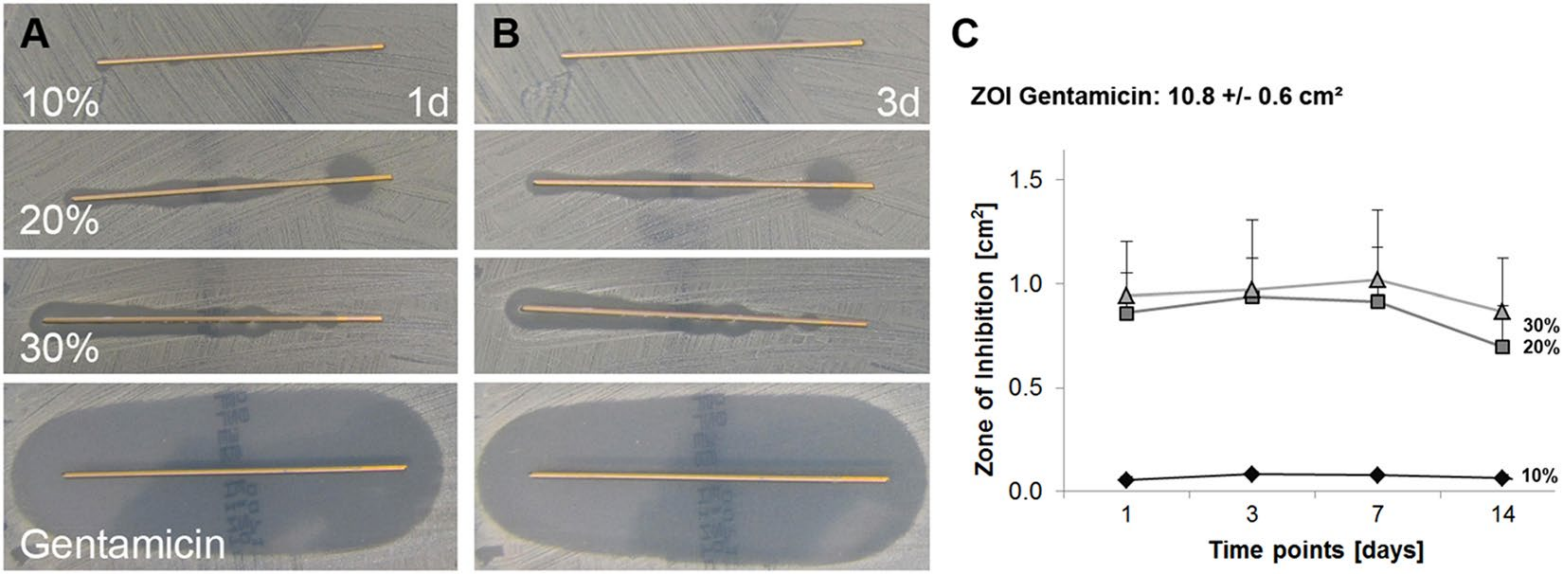
Zone of inhibition (ZOI) test of the AMP2 or gentamicin incorporated at different concentrations into a poly(D,L-lactide) coating (n = 3). Gentamicin was used in 10% w/w PDLLA, AMP2 ranged from 10, 20 to 30% w/w PDLLA. (**A**) ZOI after 1 day, (**B**) ZOI after 3 days, (**C**) Quantification of the ZOI, n = 3. Data are shown as means with standard deviation.

Performing a Zone of Inhibition test revealed that the AMP2 incorporated into the polymer-coating remained bioactive and inhibited the growth of *S. aureus* in a dose dependent manner (Fig. 8). The activity, however, was much lower compared to gentamicin, as also seen in the MIC/MIB (Fig. 1).

## Discussion

A severe complication in orthopedic surgery is the development of an implant associated infection, which is difficult to treat and in most cases the removal of the implant combined with a prolonged antimicrobial therapy is necessary^19^. The ideal antimicrobial drug in this case will be active against different bacterial strains, bacteria in a biofilm or internalized in the eukaryotic cells of the host without harming them. Cationic antimicrobial peptides seem to be a promising substance class due to their broad activity and function. The present study investigated five cationic AMPs regarding their antimicrobial activity and effect on primary osteoblast-like cells. In addition, the potency to kill bacteria in a biofilm, as a severe complication associated with materials/implants used in orthopedic surgery, was investigated. The tested AMP modifications showed antimicrobial effectivity against all tested bacteria, including 2 clinical isolates. The AMP2 was also growth inhibitory and bactericidal for a clinical MRSA isolate, which is a very important finding high lightening the potency of AMPs. The efficacy varied between the used modifications and a higher concentration compared to gentamicin was necessary (except for the MRSA) to inhibit bacterial growth and kill Gram-positive and -negative bacteria, which was also shown for other AMPs^20^. Due to the nature of the cationic AMPs, ions in the broth might cause an inhibition of the AMP^21^. Therefore, the concentration of Ca^2+^ and Mg^2+^ was varied in the broth and the effect on the AMP activity was investigated. In 80% of the experiments no or minor variations in the minimal inhibitory and bactericidal concentrations were detected for the 5 tested AMPs and the different bacteria. In 20% of the experiments the MIC or the MBC was 3 to 4 fold increased in the MH^++^ bouillon compared to the MH^−^ bouillon. The main decrease of antimicrobial activity of gentamicin or the AMPs was seen for *P*. *aeruginosa* cultured in MH^++^. This change in the MIC of gentamicin against *P*. *aeruginosa* due to the supplementation of the medium with cations was also seen in a previous study indicating the impact of cations on antimicrobial activity of substances^22^. A prerequisite for the characterization of an antimicrobial peptide is the activity of the AMP in the presence of cations, representing more physiological conditions^21^. This was demonstrated in particular for AMP2, which was active against all the tested strains including two clinical isolates in cation enriched conditions.

The used antimicrobial peptides are part of a larger optimized peptide library. The five peptides were selected based on their good broad range activity and difference in structure. They were only 9 amino acids long and only composed of inexpensive amino acids. That would allow a cost effective large scale production and therefore low extra economic burden for the health care system in case a peptide would be used routinely to prevent or cure infection of the bone. In a previous study 49 peptides of this library were used and the antimicrobial activity mainly against *Mycobacterium tuberculosis* was investigated^23^. Beside *M*. *tuberculosis* also other bacterial strains were analyzed and this and the present study showed that the optimized peptides inhibit and also kill Gram-positive and -negative bacteria. The antimicrobial activity of the used peptides, however, was unexpectedly much higher than in the present study. To confirm that not the peptide caused this difference, the MICs against the reported strains was tested again by using the same provider of Mueller-Hinton broth as described before. We could verify the previously reported MICs and therefore exclude that peptides were the source of this difference in MICs. Further studies showed that for all-L peptides indeed there can be a strong strain dependency, caused by the amount of proteases produced or changes in the envelopment of the bacteria. All-D peptides that are very stable against proteases did not show these effects (unpublished results). MH broth is also not a well-defined media and could cause the bacteria to produce more or less proteases. In addition, the pre-cultivation, and the used cultivation method might have an influence. For the AMP2 used in the detailed analysis of the present study, the MIC difference for *E*. *coli, P*. *aeruginosa, S*. *epidermidis* was approximately two fold. Whereas it was much higher for *S*. *aureus* or *E. feacalis*, respectively. In a recent publication from Mikut at al. more than 3000 9mer peptides were investigated in order to find activity rules^24^. The data confirmed previous observation that simple physicochemical parameters are not good predictors, but the right balance between R, K and hydrophobic amino acids (I, V, F, Y, and W)^25^. There is also no positional preference for any amino acid. The current set of five peptides, however, is too small to correlate activity and sequence, especially given the photolytic effects described before.

Another substance that might have an influence on the antimicrobial activity of AMPs is serum and/or components of the serum. Svenson *et al*. demonstrated that the presence of albumin induced a 10-fold increase of the minimal inhibitory concentration of lactoferricin-derivates for *Staphylococcus aureus* ^13^. It is assumed that this decrease in activity is due to the 1:1 binding of the peptide and albumin. In a further study the effect of serum on hexcameric AMPs was investigated^26^. Depending on the modification of the peptide the antimicrobial activity or the protease resistance was increased. Testing the antimicrobial activity after addition of serum, however, showed a negative effect on the activity independent of the modification. Therefore, not only the degradation in the serum might impact the activity, but also the increased ionic-concentration and also binding with serum components might be responsible. In the present study serum decreased dosage dependent the activity of the AMP and this shows the importance of testing more physiological conditions than rather optimized culture conditions. Serum concentration of 0.1 and 1% decreased slightly the activity, and allowed us to perform the cytotoxicity tests and the internalization studies with primary osteoblast.

The highest concentration of all tested AMPs inhibited significantly the viability of the osteoblast-like cells, whereas concentrations necessary for killing all the tested bacteria had no negative effect on primary human osteoblast-like cells (except for AMP5). The most antimicrobial active and less harmful peptide -AMP2- was then used for further studies. Concentrations killing the different bacteria had no negative effect on osteogenic differentiation of the osteoblast-like cells and their migratory activity. This is an important aspect, because a bony ingrowth of the implant is a prerequisite for the successful healing and the prevention of an infection and this will only happen, if the host cells win the “race for the surface”^27^. If the local cells would be impaired by the antimicrobial therapy, this osteointegration would not occur increasing the risk of infections.

During the surgical debridement the infected periprosthetic tissue should be removed and antibiotic treatment should kill all persisting bacteria. However, bacteria such as *S. aureus* and *S. epidermidis* can invade host cells and may be protected from the host immune system and antimicrobial therapy resulting in an infection at a later time point^3^. To address this possible complication the effectivity of the AMP2 reading the killing of internalized bacteria was investigated. The concentration necessary to kill the bacteria under standard conditions (67 µg/ml) inhibited significantly the bacterial load in osteoblast-like cells after 4 hours and inhibited their growth over a time-period of 24 hours. Due to the fact that this concentration had no negative effect on the cell viability and differentiation, it can be concluded that the mechanism of crossing mammalian membranes is non-damaging. This was recently also reported for modifications of cell penetrating peptides and AMPs, highlighting the potency of these two groups for new treatment strategies of intracellular infection^28^. Substitution of lysine with arginine increased the uptake of penetratin into HeLa WT cells. The substitution of arginine by lysine, however, had the opposite effect, high lightening the importance of both amino acids in this process. The AMP modification tested in the present study contains three arginine and one lysine residues, which might be responsible for the effective cell penetration seen and killing of internalized bacteria without harming the host cells. A comparison with the three other modified AMPs was not done but might show differences due to the differing ratios of arginine and lysine. However, the cell penetrating mechanism of peptides is not fully understood and might occur by direct translocation, endocytotic uptake or the formation of inverted miscelles^29^.

A further severe problem of implant associated infections is the formation of a biofilm that protects the bacteria^30, 31^. Penetration of antimicrobial active substances is affected by the biofilm and the altered susceptibility of the bacteria in the biofilm to antibiotics requires much higher dosages of substances to kill the bacteria^32–34^. Two independent approaches were used to analyze the effect of the AMP2 on biofilm. In the first approach bacteria and biofilm were visualized with a fluorescent probe directed against the ribosomal RNA that is specific for staphylococci^35^. In the second approach the energy production of the bacteria in the biofilm was measured^36^. Both methods showed that the AMP2 disturbed the biofilm (FISH) and killed the bacteria in the biofilm (microcalorimetry). The microcalorimentry data clearly showed that for eradication of the biofilm a much higher concentration was needed as for killing planktonic bacteria. The differences in the concentrations showing an effect for FISH or calorimetry can be explained on the one hand by the sensitivity of the two strains: *S. epidermidis* for FISH and *S. aureus* for microcalorimety. On the other hand by the different outcome measure: FISH visualizes the biofilm and detects changes in composition and cell count, whereas the calorimetry quantifies the heat development of cells as a measure for viability. Therefore, smaller amounts of the AMP disturb the biofilm without killing all the bacteria. For killing bacteria in a biofilm higher concentrations are needed. The two strains were chosen because they are the most relevant pathogens causing orthopedic device infections and biofilm formation. The seen differences might be caused by differences in the biofilm formation and structure between *S. aureus* and *S. epidermidis*. For example, it was shown that poly-N-acetylglucosamine surface polysaccharide (PNAG or PIA) and extracellular DNA (ecDNA) have different structural roles in *S. aureus* and *S. epidermidis* leading to different effects of degrading enzymes^37^. Similar effects to the present study were seen in a previous study using also both methods to analyze the effect of antibiotic-loaded microparticles on *S. aureus* and *S. epidermidis* biofilms^32^. Both methods showed that *S. aureus* (MRSA) biofilm was inhibited by a lower Daptomycin concentration than *S. epidermidis* biofilm. This underlines the differences of antimicrobial susceptibility of the two *Staphylococci* and also the differences in the method.

Due to the increasing development of bacterial resistance to classical antibiotics, new therapeutic approaches and alternatives to antibiotics are needed. At the moment, 19 clinical trials (Phase 1–3) for new antimicrobials including antibodies, probiotics, bacteriophages and vaccines are running^38^. How many will reach the registration and when, can only be estimated. A promising class of antimicrobial active substances are antimicrobial peptides (AMPs). Two AMP-like structures are approved for human use (Polymixin, Daptomycin) to treat skin infections and as reserve antibiotics, but three further AMPs failed FDA approval^39^. At the moment, for two AMPs clinical phase 1 or 2 studies are running^38^. Within the last years the US and European agencies (FDA and EMA) realized the urgent need for new antimicrobials and changed their guidelines to encourage the development and approval of new substances^40^. Taking the results of the present study together, the investigated AMP modifications showed an antimicrobial activity against Gram-positive and -negative bacteria at concentrations that have no negative effect on viability, differentiation and migration of osteoblast-like cells. Higher concentrations killed internalized bacteria and bacteria within a biofilm. A preliminary release study proved the possibility to incorporate the AMP into a polymer coating for local drug release from an implant. The local release from the implant coating has several advantages: release of the active drug at the site of need, controlled release over a certain time period, reduction in applied drug concentration, and reduction of systemic drug load. The great advantage of the used drug delivery system compared to other experimental systems is the CE certification. Since 2005 this coating is used on intramedullary tibia nails loaded with gentamicin for infection prophylaxis^41^. But several other coating techniques are investigated in preclinical studies^42–45^. To combine the antimicrobial treatment with defect regeneration or in prosthetic surgery, other applications are also possible such as perioperative mixing of the AMP with bone grafting material, such as autologous or allogeneic bone, polymers, tricalciumphosohate, bioglass or cement^46, 47^.

Focusing on the prevention and treatment of implant associated infections in orthopedics, the results of the present study are promising because the main obstacles are addressed: killing of planktonic, biofilm and internalized bacteria without impairing the function of host cells, allowing an osteointegration of the implant. Based on these encouraging results further optimization can provide researchers in academia and industry with lead compounds to specially address implant associated infections.

## Material and Methods

### Test substances

**Figure 9:**
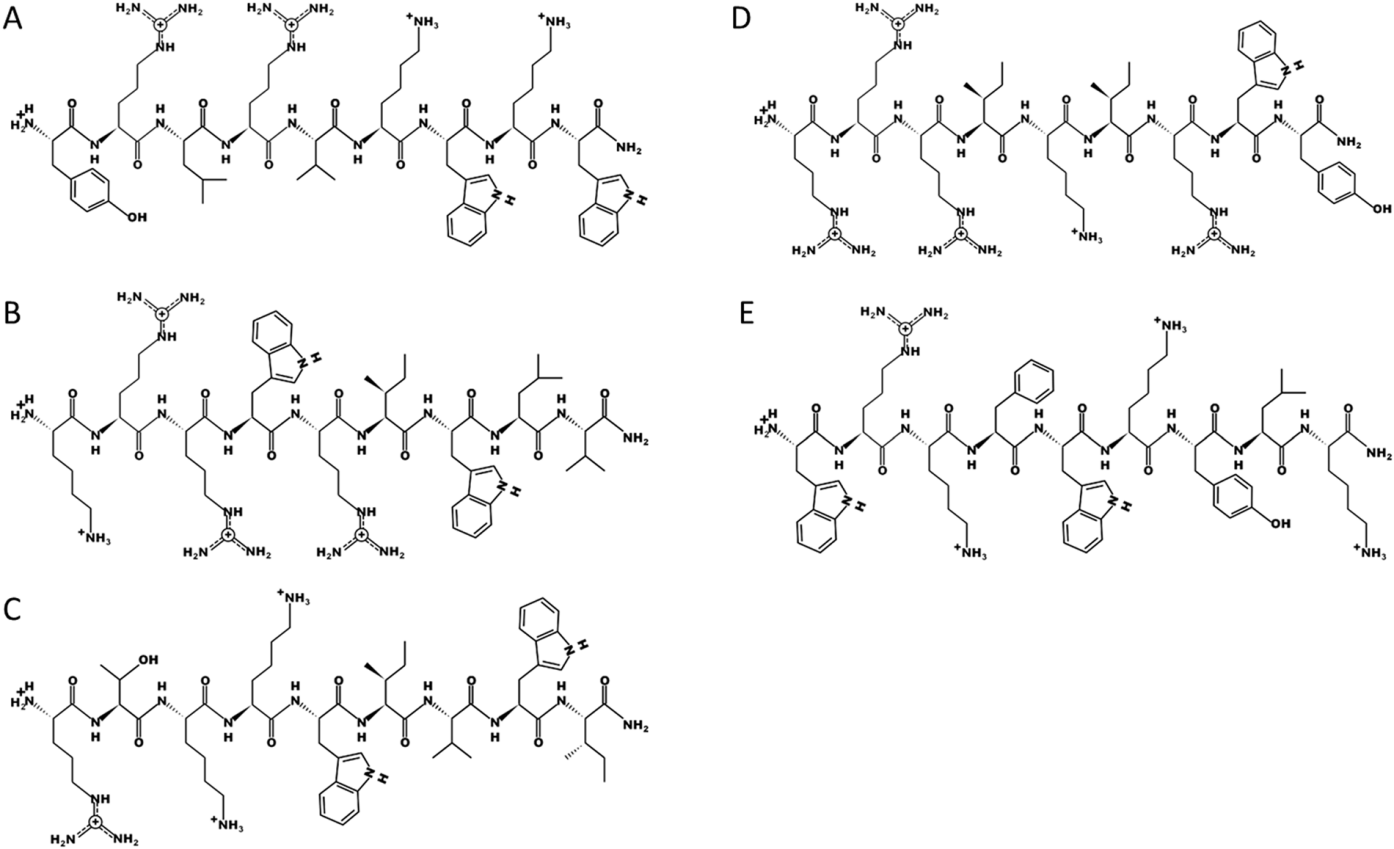
Two dimensional drawing of the five selected peptides using the PepDraw online software. (**A**) AMP1, (**B**) AMP2, (**C**) AMP3, (**D**) AMP4 and (**E**) AMP5.

Cationic Antimicrobial Peptides (AMP) from an optimized peptide library were prepared using a solid-phase peptide synthesis procedure (SPPS). Briefly, SPPS was performed on a 50 μM scale using a multiple peptide synthesizer (Intavis, MultiPep RS, Tuebingen, Germany) and a standard Fmoc/t-Bu protocol, comprising double coupling at every cycle. Raw peptides were purified via RP-HPLC (peptide purity used in biologic assays was higher than 95%) and concentration was determined with NanoDrop 1000 UV/VIS spectrophotometer (Thermo Fisher Scientific, USA). Five peptides from the optimized peptide library were initially used (Table 2, Fig. 9).

**Table 2:** Information on tested antimicrobial peptides.

Name	Sequence^[1]^	MW^[2]^	Charge^[3]^	IP [pH]	GRAVY^[4]^	composition^[5]^
AMP1	YRLRVKWKW	1338.64	5	12.2	−1.322	π+α+α+π+π
AMP2	KRRWRIWLV	1316.64	5	14	−0.744	+++π+απαα
AMP3	RTKKWIVWI	1232.54	4	14	−0.178	+ο++πααπα
AMP4	RRRIKIRWY	1351.67	6	12.6	−0.678	+++α+α+ππ
AMP5	WRKFWKYLK	1358.67	5	11.7	−1.411	π++ππ+πα+

^[1]^N-term: free amine; C-term: carboxamide, ^[2]^Molecular weight [g/mol], ^[3]^Net charge at pH 7 ^[4]^Grand average of hydrophathicity (calculated using online software http://web.expasy.org/protparam)^[5]^+ basic; ο polar;π aromatic; α aliphatic.

All C-termini (R-COO-) of peptides were amidated by ammonia to carboxamide groups (R-CO-NH2) and as a result, the former negatively charged C-terminus is turned to neutral. This enhances the overall positive charge of the antimicrobial peptides, which is one of the critical points for antimicrobial activity. Depending on the experiment, different antibiotics were use as reference: Gentamicin sulphate powder (~60% pure gentamicin, 477.6 Da; Fujian Fukang Pharmaceutical Co., Ltd., China); Eremfat (pure rifampicin, 822.9 Da; Riemser, Germany) and Cubicin (pure daptomycin, 1619.7 Da; Novartis Pharma GmbH, Switzerland).

### Bacterial strains

The strains *Escherichia coli (*ATCC 25922), *Enterococcus faecalis* (ATCC 29212), *Pseudomonas aeruginosa* (ATCC 27853), *Staphylococcus aureus* (ATCC 29213) were used. In addition, following clinical isolates were used: *Staphylococcus epidermidis* (PIA 8400, isolated from blood cultures^48^), and two *Staphylococcus aureus* (MRSA and MSSA, both isolated from osteosyntheses material, kindly provided by Andrej Trampuz, Charité).

### Minimal Inhibitory Concentration and Minimal Bactericidal Concentration

To determine the minimal inhibitory concentration (MIC), classical broth microdilution method was performed after protocols from Wiegand *et al*.^15^. The influence of cations was analyzed by using cation adjusted Mueller-Hinton bouillon II with 20–25 mg/l Ca^2+^ and 10–12.5 mg/l Mg^2+^ (MH^++^; BBL, Germany) or Mueller-Hinton bouillon without cations (MH^−^; Merck, Germany) (n = 4–8).

The maximal concentration of the AMPs was between 200–271 µg/ml. The differences in the AMP concentration are due to the purity of the peptides (absorption coefficient from 5.19 to 9.36). A bacterial inoculum of 1–5 × 10^5^ colony forming units (cfu)/ml was incubated at 37 °C in an aerobe atmosphere (*E. faecalis* with 5% CO_2_) for 18–24 hours and the MIC was defined as the concentration without visible growth.

To identify the minimal bactericidal concentration (MBC), 80 µl of two wells with the determined MIC and two concentrations above this were plated on Columbia-agar (CA) plates with 5% sheep blood and incubated overnight at 37 °C.

In addition, the influence of fetal calf serum (FCS, containing 17 g/l albumin; Biochrom, Germany) on AMP activity was determined by preincubating 67 µg/ml of the AMP2 with 0–10% FCS at 37 °C for 2 hours followed by incubation with *S. aureus* for 3 hours. The control group, without AMP, was FCS free (n = 3).

### Cytotoxicity, differentiation and migration

Human primary osteoblast-like cells were isolated from human femoral head with permission of the local ethic committee. The patients gave their written informed consent. The tissue harvest was carried out in accordance with the local guidelines of the Charité-Universitätsmedizin Berlin and the study was approved by the ethics committee of the Charité-Universitätsmedizin Berlin (EA4/035/14).

(1) For the cytotoxicity test, 1 × 10^4^ cells were seeded in 48-well plates with cultivation medium (CM) DMEM/Ham’s F-12 (D/H; Biochrom, Germany) + 10% fetal calf serum (FCS; Biochrom, Germany) + 1% penicillin/streptomycin (P/S; Biochrom, Germany) + 0.05 mM L-ascorbic-2 phosphate (L-Asc) + 0.05 mM β-glycerolphosphate (β-Glyc) and incubated overnight at 37 °C, 5% CO_2_ and 95% relative humidity. After adding the AMPs (3–542 µg/ml - depending on the AMP) or gentamicin (8–1024 µg/ml) in CM with 1% FCS, without Pen/Strep, cells were cultured for three days. Cell viability was determined with an AlamarBlue® assay before adding the AMPs and after 3 days. Alkaline phosphatase activity was measured on day 3. Cells from two donors were used and the experiments were done in triplicate (n = 6).

(2) The effect of the most antimicrobial active and less toxic AMP (AMP2) on osteoblast differentiation was investigated. Confluent cells were cultured for 21 days in cultivation medium (CM), differentiation medium (DM: D/H + 10% FCS + 1% P/S + 0.5 mM L-Asc + 10 mM β-Glyc + 100 nM dexamethasone + 1.5 mM calcium chloride) and DM with a low (17 µg/ml) and a high (133 µg/ml) AMP2 concentration. Medium was changed every 3–4 days and fresh AMP was added by each change. Cells from two donors were used and the experiments were done in triplicate (n = 6).

#### Cell viability

Alamar Blue® (Biozol, Germany) was done according to the manufacturer’s instruction.

#### Alkaline phosphatase (AP) activity

Cells were incubated with 0.13% w/v 4-nitrophenyl phosphate disodium salt hexahydrate (Sigma-Aldrich, Germany) dissolved in 0.1 M AP-buffer (50 mM glycine, 100 mM Tris-Base, 1 mM MgCl_2_ in dH_2_O) at pH 10.5 for 30 min at 37 °C and absorbance was measured at 405 nm wavelength.

#### Alkaline phosphatase staining

Cells were fixed with 3.7% v/v formalin for 10 min at room temperature (RT) and stained with 0.1% w/v naphtol-AS-MX-phosphate (Sigma-Aldrich, Germany) and 0.6% w/v Echtblausalz (Chroma, Germany) in staining buffer (0.5% dimethylformamide, 100 mM Tris-Base, 2 mM MgCl_2_ in dH_2_O; pH 8.5) for 30 min at 37 °C.

#### Alizarin Red staining and quantification

Cells were fixed with 3.7% v/v formalin for 10 min at RT and incubated with 0.5% w/v alizarin red (Sigma-Aldrich, Germany) in dH_2_O for 10 min at RT. Quantification was done by removing staining with 5% w/v SDS in 5 mM HCl and measuring absorbance at 570 nm.

#### Gene expression

RNA was isolated after 0, 7, 14 and 21 days in culture using the NucleoSpin RNA Kit (Macherey Nagel, Germany). The RNA of three wells was pooled resulting in n = 2. RNA (100 ng) was transcribed into complementary DNA (cDNA, qScript cDNA Supermix, Quanta BioSciences, USA) and used 1:20 diluted. RT-PCR analysis were run with validated primer sets (for primer sequences see Table 3) and PerfeCTa® SYBR® Green SuperMix (Quanta BioSciences, USA). For all primers, amplification efficiencies were analyzed and mean normalized expression (MNE) ratios were calculated using the ΔΔCt method with efficiency correction and normalization to the control (d0 of differentiation) and 18 S ribosomal RNA used as a reference gene.

**Table 3:** All primer pairs were designed with the Primer3web web page and intron spanning.

Gene	Name	Efficiency	forward (F) and reverse (R) sequence 3′-5′
18 S	18 S	1.79	F: CGG AAA ATA GCC TTT GCC ATC
			R: AGT TCT CCC GCC CTC TTG GT
alkaline phosphatase	AP	1.81	F: GGA AAT CTG TGG GCA TTG TG
			R: CCC TGA TGT TAT GCA TGA GC
collagen type I, alpha 1	Col1	1.92	F: TGA CCT CAA GAT GTG CCA CT
			R: ACC AGA CAT GCC TCT TGT CC
runt-related transcription factor 2	RUNX2	1.86	F: GCC CCC AAA CAG TAT CTT GA
			R: GCC TGA AGT GAG GTT TTA GGC

Using the OligoAnalyzer 3.1 software all primer pairs were tested for self-dimers, hetero-dimers or hairpin production. The amplicon size was chosen between 150–250 base pairs.

(3) The effect of AMP2 on cell migration was assessed. Human primary osteoblast-like cells were seeded into 24-well plates and cultured in cultivation media. After reaching confluence, medium was removed and a cross scratch was made using a 10 µl pipette tip using a modified protocol^49^. Medium plus AMP2 (2–133 µg/l), 0.1% FCS (negative control) and 10% FCS (positive control) was added and pictures were taken at time point 0, 24 and after 48 hours of cultivation. Using ImageJ software (version 1.4.8.; https://imagej.nih.gov/ij/) the diameter of the scratch was measured and the closure over time calculated (n = 3).

### Effect against internalized bacteria

Human primary osteoblast-like cells were cultivated in 24-well plates with cultivation medium until they reached a minimum confluence of 80%. For the experiment, cells were washed and *S. aureus* (ATCC 29213) in cultivation medium added (~2 × 10^7^ cfu/ml). After an incubation of 45 minutes, extracellular bacteria were killed using 200 µg/ml gentamicin dissolved in CM with 10% FCS, without P/S. After 1 hour incubation, gentamicin solution was removed, cells washed three times and AMP2 (33 and 67 µg/ml) in CM with only 1% FCS + no P/S added for 4 and 24 hours. As reference, 7.5 µg/ml rifampicin was used. All incubation steps were done in an incubator with 37 °C, 95% humidity and 5% CO_2_. For determination of the internalized bacteria, cells were lysed and the supernatant plated on CA plates. Experiment were done with n = 7.

### Biofilm

Fluorescence *in situ* hybridization (FISH) was used to visualize the effect of the AMP2 on a biofilm^35^. *S. epidermidis* (PIA 8400) biofilm was grown on 5 × 8 mm polyurethane (PU) waver (Epurex Films, Germany) in the incubator at 37 °C for 72 hours under aerobic conditions. After biofilm formation, medium was replaced by MH^++^ medium containing different concentrations of the AMP2 (4 or 8 µg/ml) or phosphate buffered saline as control. After further 24 hours of incubation on a rotation shaker, waver were removed, fixed and embedded in methylmethacrylate (Technovit 8100, Kulzer, Germany) and sectioned as described elsewhere^50^. To visualize the biofilm, samples were stained with nucleic acid stain DAPI and the Cy3-labeled *Staphylococcus-*specific FISH-probe STAPHY (5′TCCTCCATATCTCTGCGC3′, biomers, Germany) as published^51, 52^. A nonsense probe NON338 labeled in Cy5 was used to exclude unspecific probe binding^53^. After incubation in a dark, humid chamber for 2 hours at 50 °C, the slides were rinsed with sterile water, dried, and mounted using Vectashield mounting medium (Vector Laboratories, Burlingame, CA). Image analysis was done using an epifluorescence microscope (Axioplan 2; Carl Zeiss, Jena, Germany) equipped with narrow band filter sets (AHF Analysentechnik, Germany). Image acquisition was performed with an AxioCam MRm (Zeiss, Germany) making use of the AxioVision 4.4 software. Digital image analysis was performed with the daime software^54^. Per group 10 pictures were randomly chosen and analyzed.

### Calorimeter

To confirm MIC of the AMP2 by isothermal microcalorimetry^32^, three different bouillons were tested: Mueller-Hinton II bouillon with adjusted cations (MH^++^; BBL, Germany), Mueller-Hinton bouillon with low cations (MH^+^; with 3–4 mg/l Ca^2+^ and 5–6 mg/l Mg^2+^; Oxoid, Germany) and Mueller-Hinton bouillon without cations (MH^−^; Merck, Germany). AMP2 concentrations of 2–33 µg/ml were tested with the three different bouillons and gentamicin concentrations of 0.125–2 µg/ml were analyzed with MH^++^. *Staphylococcus aureus* (ATCC 29213, 300 µl with 10^5^ cfu/ml) was added to each microcalorimetry glass ampoule (Waters; Germany) containing 3.7 ml of AMP or gentamicin solution of (n = 3). The ampoules were hermetically sealed and heat flow (W) was measured for 24 hours in the calorimeter TAM III (TA Instruments, USA). For the biofilm assay, *S. aureus* biofilm was grown on glass beads (4 mm diameter, 60 µm pores; ROBU Glasfilter-Geräte, Germany) in MH^++^ bouillon at 37 °C in an aerobe atmosphere for 24 hours. After washing with sodium chloride, beads were incubated with AMP2 (133, 267, 533 µg/ml) or daptomycin (128 µg/ml) in MH^++^ bouillon for another 24 hours at 37 °C. Beads were washed again and added to the glass ampoule with fresh MH^++^ bouillon. The total heat (J) was measured for 48 hours in the calorimeter. A concentration, which inhibits minimum 90% of biofilm, can be defined as the minimal biofilm inhibitory concentration (MIC_B_). All samples were tested with n = 6.

Furthermore, the MBC for biofilms (MBC_B_) where 99.9% of all bacteria are killed was determined. After measurement, bouillon and glass beads were plated on CA plates and incubated for 24 hours at 37 °C in an aerobe atmosphere.

### AMP coating of implants

Titanium wires (diameter 1 mm, Synthes, USA) were coated with Poly(D,L-lactide) (PDLLA, Boehringer Ingelheim, Germany) and incorporated AMP2 at three different concentrations: 10% w/w, 20% w/w, or 30% w/w. Gentamicin/PDLLA (10% w/w) or PDLLA coated wires served as control. Briefly, 100 mg PDLLA was solved in 1.5 ml ethyl acetate and the wires were coated in a dipping procedure. Details of the coating were described previously^55^. Three nails of each group were placed on Mueller-Hinton agar plates plated with *S. aureus* (ATCC 29213) and the zone of inhibition (ZOI) was quantified after 1, 3, 7, and 14 days using ImageJ software.

### Statistical analyses

The data were analyzed with the non-parametric tests Kruskal-Wallis and Mann-Whitney (SPSS 22; IBM, USA). The multiple alpha-error was corrected with Bonferroni-Holm post-hoc test. Values with p ≤ 0.05 were considered as significant. The effect of FCS concentration on AMP activity was analyzed by linear regression.
